# Is circumferential urethral mobilization an overdo? A prospective outcome analysis of dorsal onlay and dorso - lateral onlay BMGU for anterior urethral strictures

**DOI:** 10.1590/S1677-5538.IBJU.2016.0599

**Published:** 2018

**Authors:** Gaurav Prakash, Bhupendra Pal Singh, Rahul Janak Sinha, Ankur Jhanwar, Satyanarayan Sankhwar

**Affiliations:** 1 Department of Urology, King George's Medical University, Lucknow, India

**Keywords:** Urethral Stricture, Lower Urinary Tract Symptoms, Buccal mucosa

## Abstract

**Introduction:**

For dorsal onlay graft placement, unilateral urethral mobilization is less invasive than standard circumferential urethral mobilization. Apart from success in terms of patency of urethra, other issues like sexual function, overall quality of life and patient satisfaction remain important issues while comparing outcomes of urethroplasty.

**Aim:**

To prospectively compare the objective as well as subjective outcomes of two approaches.

**Materials and Methods:**

Between July 2011 and January 2015, 136 adult males having anterior urethral stricture with urethral lumen ≥ 6 Fr. were prospectively assigned between two groups by alternate randomization. Operative time, complications, success rate (no obstructive symptoms, no need of any postoperative intervention, Q max > 15mL/sec), sexual functions (using Brief Male Sexual Function Inventory) were compared.

**Results:**

Baseline parameters were similar in both groups (68 in each group). Overall success rate was similar in both groups (89 % and 91 % respectively). Improvement in total LUTS scores was similar in groups. Changes in overall health status (VAS and EQ 5D) was equal in both groups. Erectile function score was significantly decreased in DO than DL group while ejaculatory function and sexual desire remained stable after urethroplasty in both groups.

**Conclusions:**

In anterior urethral stricture buccal mucosa graft provides satisfactory results as onlay technique. No technique whether dorsolateral and dorsal techniques is superior to other. Dorsolateral technique needs minimal urethral mobilization and should be preferred whenever feasible.

## INTRODUCTION

Buccal mucosa for substitution urethroplasty was initially used in the late 1980s. It became an ideal urethral substitute since it is a hairless graft, ease to harvest, compatible with a wet environment, easy to surgical handling and with a good graft survival. Buccal mucosa has been used as substitute in various techniques, including ventral onlay, and dorsal onlay procedures (1), ventral sagittal urethrotomy with inlay patch, (2, 3) and one side urethral mobilization (4, 5). Barbagli dorsal onlay technique of circumferential urethral mobilization has been widely used with success rate of approximately 99% and 66% in the short and long-term follow-up, respectively (6). But issues have been raised regarding risks of compromised vascularity of the urethra due to complete separation from corporal bed during dissection. It becomes a major concern particularly if the meatus is also involved with disease and the most distal urethra is extensively dissected.

Inlay patch by ventrally incising the urethra is another option for substitution urethroplasty. It preserves the vascularity of the urethra, but due to narrow urethral plate, graft width is compromised (2).

Dorsolateral onlay urethroplasty by unilateral urethral mobilization is another technique that has been used with success results around 80-90% in various studies (4). By one side mobilization, the urethral vascularity of urethra is maintained by keeping the graft in a dorsolateral onlay fashion. Technically, it is as easy as Barbagli procedure and reported short terms results have been encouraging (4). It also preserves the native one sided blood supply at meatus as well as distal urethra. By using this approach, at least the one-sided bulbo-spongious muscle remains intact along with ipsilateral vascularity to the urethra.

In relation to superiority, there are very few studies comparing various techniques of urethroplasty (7, 8). In our center, we conducted a prospective randomized study to find out the results of dorsal and dorsolateral technique in anterior urethral stricture in terms of voiding function, patient's quality of life and sexual function. The aim was to prospectively compare the objective as well as subjective outcomes of dorsal and dorsolateral onlay urethroplasty.

## MATERIALS AND METHODS

This study was conducted at the department of Urology, King George's Medical University, Lucknow, with proper ethical clearance by institutional review board. Between 2011 and 2015, a total of 136 adult males with diagnosis of anterior urethral stricture with minimal urethral lumen of 6Fr were prospectively assigned to two Groups by alternate randomization (1:1). Each group included a total of 68 patients that were submitted to dorsal or dorsolateral onlay urethroplasty. Those with active urinary tract infection, uncontrolled diabetes mellitus, history of previously failed urethroplasty and lichen sclerosis were excluded from the study.

Detailed history including duration of symptoms, history of catheterization, and any previous endoscopic intervention was taken. Personal history of smoking and tobacco chewing was also obtained. Patients were locally examined to assess urethral meatus, urethral consistency and any BXO (balanitis xerotica obliterans) changes. Mouth was examined for health of oral mucosa.

Pre-operative lower urinary tract symptoms and sexual function were noted by standardized questionnaire PROM (patient reported outcome analysis), and BMSFI (Brief male sexual function inventory) score, respectively (9, 10).

PROM questionnaire consists of six summative questions (Q1-6) for lower urinary tract symptoms (LUTS), a separate LUTS-specific quality-of-life (QoL) question (Q7) and voiding picture (Q8). VAS (visual analogue score) was also measured to know the improvement of subjective symptoms and was calculated on the scale of 0 to 100. BMFSI is an 11-item standardized, validated questionnaire to assess the key dimensions of sexual response. In this study, seven questions were used from the BMSFI to evaluate Ejaculatory Function (two questions), Sexual Desire (two questions) and Erectile Function (three questions).

Each patient was evaluated with routine clinical investigation and uroflowmetry. Retrograde urethrogram (RGU) and voiding cystourethrogram (VCUG) of each patient was done to stablish the site and length of stricture.

Data were entered in the MS-Excel computer program and SPSS 16.0 software was used for analysis. Paired t test was used to detect significance from baseline value to follow-up time in case of continuous variables and unpaired t test was used to detect the differences between two continuous variables. A p value less than or equal to 0.05 was considered statistically significant.

### Operative procedure

Before surgery, every patient underwent urethroscopy to assess stricture length and location, and calibre of urethra. Only those patients who had urethral lumen superior to 6Fr proceeded to urethroplasty. Urethra was exposed through a midline perineal incision.

### Dorsal onlay urethroplasty

Urethra was mobilized circumferentially and then rotated by 180 degrees and opened along its dorsal surface. Then, harvested buccal graft was placed over the corpora dorsally and proper quilting was done using polygalactin 4-0 sutures. Cut edge of the urethra was sutured with graft material and a 14Fr silicon catheter was placed.

### Dorsolateral onlay urethroplasty

In this surgical technique, unilateral mobilization of the urethra was done. Urethral incision was extended beyond stricture for about 1cm both proximally and distally to the normal urethra. The proximal and distal urethral lumens of the urethra were calibrated/scoped. In all the cases, graft was sutured with the urethral margin meticulously, using 4-0 polygalactin suture and a14Fr silicon catheter was placed. Few interrupted stitches were used to fix the graft to the corpora cavernosa dorsally. After achieving hemostasis, wound was closed in layers. Suprapubic catheter if present was changed to a new one and remained in situ.

### Follow-up

Patients were kept on antibiotics for 5-7 days. Six to eight weeks after the operation, the urethral catheter was removed. In case of doubtful healing/fistula, pericatheter RGU was done and if present, catheter was kept for a few more weeks. Any preplaced SPC was removed on successful voiding trial. Patients were followed up at 3, 6 and 12 months and every 6 months thereafter. During follow-up, patients were clinically evaluated. PROM and BMFSI questionnaire were applied in each follow-up to find out subjective improvements following urethroplasty. Objective assessment of urinary flow was done with uroflowmetry at specified follow-up.

The primary endpoint of this study was to determine the success after each urethroplasty. Success of urethroplasty was defined as maximum flow rate >15mL/sec, urethral calibre >12Fr, no obstructive symptoms, no stricture found on RGU and no intervention (DVIU or coaxial dilatation) was needed until the last follow-up.

## RESULTS

**Figure 1:**
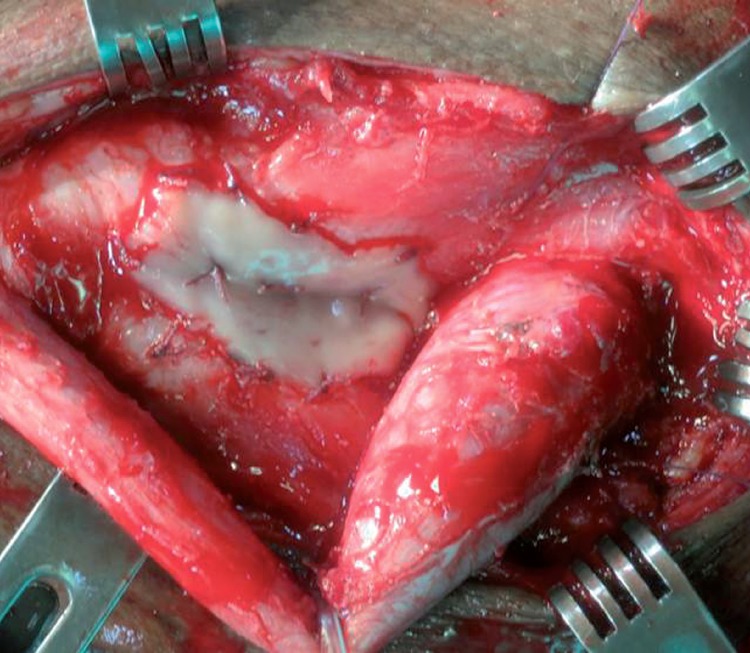
Dorsal onlay technique.

**Figure 2:**
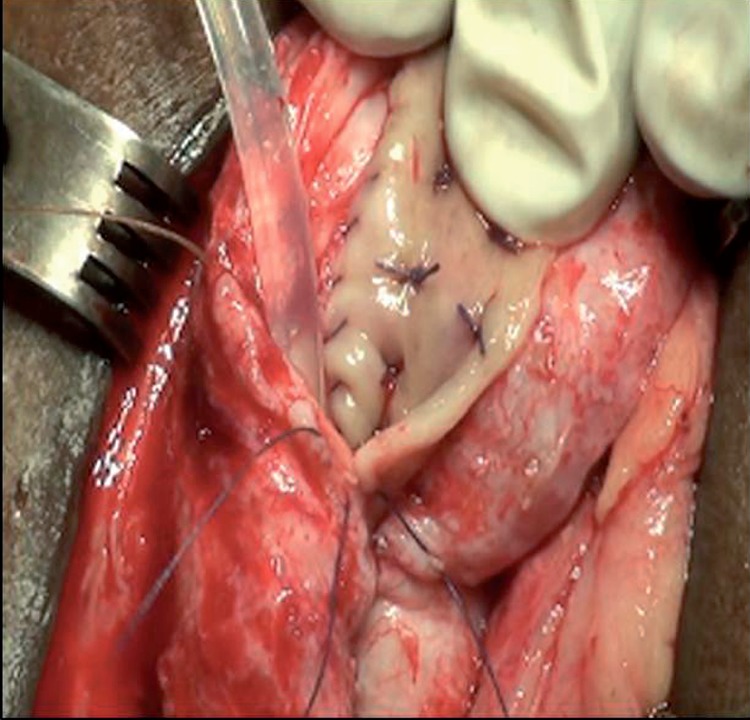
Dorsolateral technique.

Demographic data and results are shown in Tables 1 and 2. Dorsal onlay urethroplasty (Figure-1) was considered as Group-1 and dorsolateral onlay urethroplasty as Group-2. Baseline parameters were similar in both the Groups. Out of 136 patients who were included, 68 patients were included in each Group of onlay buccal graft urethroplasty and completed at least 12 months of follow-up. Mean follow--up was 28.66±12.26 months in first group and 28.22±14.42 months in second Group (Figure-2).

**Table 1:** Basic demographic data.

Parameter (mean±SD)		Group1 (n=68)	Group 2 (n=68)	P value
Age (years)		33.16±12.36	28.16±14.12	0.240
Symptoms duration (months)		43.23±25.32	47.31±23.57	0.8438
Previous-intervention		27	31	1.00
**Stricture location**				
	Penile	11	14	0.5820
	Bulbar	18	19	1.375
	Peno-bulbar	39	35	0.6563

**Table 2:** Follow-up.

Parameter (mean±SD)		Group 1	Group 2	P value
length (cm)		8.47±1.65	9.25±2.11	0.144
Operative time (min)		152.10±25.51	143.32±17.43	0.3431
Success rate (%)		89	91	0.89
Difference in Qmax (pre and post op)		12.58±6.22	11.82±4.26	0.7520
Change in LUTS		10.62±4.86	11.91±3.39	0.1570
Improvement in LUTS specific QoL		1.17±0.70	1.63±1.14	0.9474
**Health status**				
	VAS	29.21±7.34	31.56±6.92	0.69
	EQ5D	0.0772±0.163	0.780±0.186	0.57
**Sexual function**				
	Desire	0.24±0.96	0.31±0.43	0.94
	Erection	2.11±1.28	0.8±1.41	0.03
	Ejaculation	0.82±1.05	0.54±0.91	0.73

Baseline parameters were similar in both Groups. Mean duration of surgery and hospitalization were similar, with mean operative durations of 152.10±25.51min and 143.32±17.43 min in Groups 1 and 2, respectively, and 7.21±3.41 days and 7.46±3.66 days, respectively.

Mean Qmax in both Groups showed significant improvement after surgery. In Group-1, mean Qmax improved by 12.58±6.22mL/min while in Group-2, 11.82±4.26mL/min. Within the Groups, improvement seen in mean Q max was found to be statistically significant (P<0.001) but statistically insignificant (P=73) between the Groups.

Mean change in LUTS score was 10.62±4.86 and 11.91±3.39 respectively in Groups 1 and 2 and was statistically significant (P=0.013).

Similarly, mean change in quality of life score (Q7) was 1.63±1.14 in Group 1 and 1.23±1.08 in Group 2, that was statistically significant (p<0.01). Mean change in sexual desire and ejaculatory function was not found significant (P=0.94 and 0.73 respectively) but the mean change in erectile function was significant (p=03).

During follow-up in Group-1, seven patients failed. Four developed recurrence at the junctional sites (2 at distal and 2 at proximal), and all of them were managed with urethral dilatation/direct visual internal urethrotomy. One patient developed meatal stenosis which was managed by metal self-calibration. The other two patients needed lay opening of urethra.

In Group-2, six patients failed. Five patients developed anastomotic stricture and were managed by dilatation/DVIU. Out of which, one patient underwent redo urethroplasty by lingual graft urethroplasty at later stage. One patient needed perineal urethrostomy and underwent redo urethroplasty later on.

The overall success rate of dorsal onlay and dorsolateral onlay urethroplasty was 89% and 91%, respectively.

There were minor complications (Table-3) in both the Groups and oral site morbidity was minimal. There were 3 patients in Group-2 who had altered oral sensation during eating within 6 months, that gradually improved. In the long term, no patients had salivary gland infection or trismus.

**Table 3:** Complications.

Complication	Group 1	Group 2
Superficial wound infection	3 (4.41%)	2 (2.94%)
Post-void dribbling	Nil	2 (2.94%)
Penile curvature	1 (1.47%)	2 (2.94%)
Decreased genital sensation	3 (4.41%)	4 (5.88%)
Urethral stricture recurrence	7 (10.2 %)	6 (8.82%)

## DISCUSSION

Surgical treatment of anterior urethral stricture diseases is still evolving and one stage substitution urethroplasty remains the mainstay of treatment. There are various techniques for buccal mucosa graft urethroplasty, but no single technique is preferred. Until the present, most used worldwide techniques for BMGU is dorsal onlay technique due to better support for the graft as well as good vascular bed that is provided particularly by penile urethral strictures. Complication rates are also lower with lesser post void dribbling in comparison to ventral onlay urethroplasty (7). Short term and long term success rates have been very satisfactory.

To avoid the extensive circumferential mobilization of the urethra, one-sided urethral mobilization using dorsolateral technique may be a good alternative. Dorsal substitution urethroplasty has limitations in patients of multiple DVIU or more proximal stricture, while dorsolateral graft placement can be done even in strictures higher up in the urethra. In the dorsolateral technique, the policy of limited urethral mobilization remains the key to maintain the urethral vascularity on one side of the urethra.

After comparing the results, both techniques had similar success rates (89% and 91%, respectively, in dorsal and dorsolateral Group) but the easiest technique was dorsolateral graft placement.

There are few studies that have compared various onlay techniques of graft urethroplasty. A retrospective study was done by Barbagli et al. including ventral, dorsal and lateral approach. Results were similar with different techniques (7). But a recent study by Singh et al. showed better results with inlay patch urethroplasty in comparison to circumferential urethra mobilization, stressing the need for minimal mobilization of urethra (11).

In the present study, despite of long segment strictures, success rate of both the techniques were encouraging. Results of dorsolateral graft placement with lesser urethral mobilization provided similar results. The procedures were easily done with similar mean operating time of approximately 140-150 min. The average length of hospital stay was similar in both Groups.

Recurrence rates were approximately 10% in both Groups at a mean follow-up of approximately 28 months. Most of the patients who had recurrence presented alterations at the anastomotic site, either at proximal or distal to anastomosis, which were managed endoscopically. Only few patients required another open surgery. Various other studies have also shown similar recurrence rates in their short term follow-up (12-15).

In this study, a validated questionnaire of PROM was used. Patient satisfaction was similar in both Groups in terms of quality of life. Sexual satisfaction rate was assessed by another BMSFI questionnaire and was similar in both Groups, except for erectile function. The erectile function score was found better in dorsolateral group than dorsal Group. The improvement in erectile function could be due to lesser mobilization of urethra from the corporal bed. There is no comparison of this kind in previous studies as no other comparison between dorsolateral technique and others. We consider one side urethral mobilization could be one of the factors responsible for better erectile function in dorsolateral Group. Kulkarni et al. in his study came up with the suggestion that muscular and neurogenic support for urethra remains undisturbed while using minimal mobilization, and this can be one of the reasons behind better erection. Although further studies of this kind are necessary to ascertain the role of minimal mobilization in preserving erectile function, we considered this a good technique in younger patients who are concerned with surgical outcome in terms of erectile function (4). Barbagli (7) cautioned regarding dorsal access, since it might damage erectile function and the bulbar arteries when the dissection from the corpora was proximal.

Complication rates were minimal in both Groups. Post-void dribbling was more commonly found in ventral onlay techniques due to the chance of sacculation formation. These were not found in dorsal onlay Group, while only 2 patients had post-void dribbling in dorsolateral Group. The reason behind this appears to be graft adherence to a defined tissue which allows minimal sacculation formation. Most stricture recurrence were observed at anastomotic site. Specific reason cannot be cited reliably but less vascularity at distal sites could be one of the reason due to which graft integration was not good. Barbagli et al. also cited similar reason behind the recurrence (7). While proximal recurrence could be caused by a non-meticulous mucosa-to-mucosa suturing, more studies are required to ascertain the causes (7).

Several limitations may be pointed out. This was a single center study and surgeries were done by more than one surgeon experienced in dealing with urethral strictures. Use of tobacco and health of oral mucosa were not considered as factors for outcome, but these factors are known to influence the outcome (16). The mean follow-up period of 28 months is a short time to draw definite conclusions. Although the complication mentioned here occurred at shorter follow-up, a longer follow-up is necessary to comment upon late complications, such as urethral diverticula. Similarly, erectile function have been seen to be reversible, so it is necessary to stablish how much time it takes to come to normal, what requires a longer follow-up.

## CONCLUSIONS

In anterior urethral stricture, buccal mucosa graft provides satisfactory results in onlay techniques. Both dorsal onlay and dorsolateral onlay techniques provide similar success. Dorsolateral technique needs minimal urethral mobilization and should be preferred whenever feasible.
